# Supplementary figures and images for: Comparison of Protective Effects of Antidepressants Mediated by Serotonin Receptor in Aβ-Oligomer-Induced Neurotoxicity
Source: Biomedicines. 2024 May 23;12(6):1158. doi: 10.3390/biomedicines12061158 (PMC11200737; doi:10.3390/biomedicines12061158)

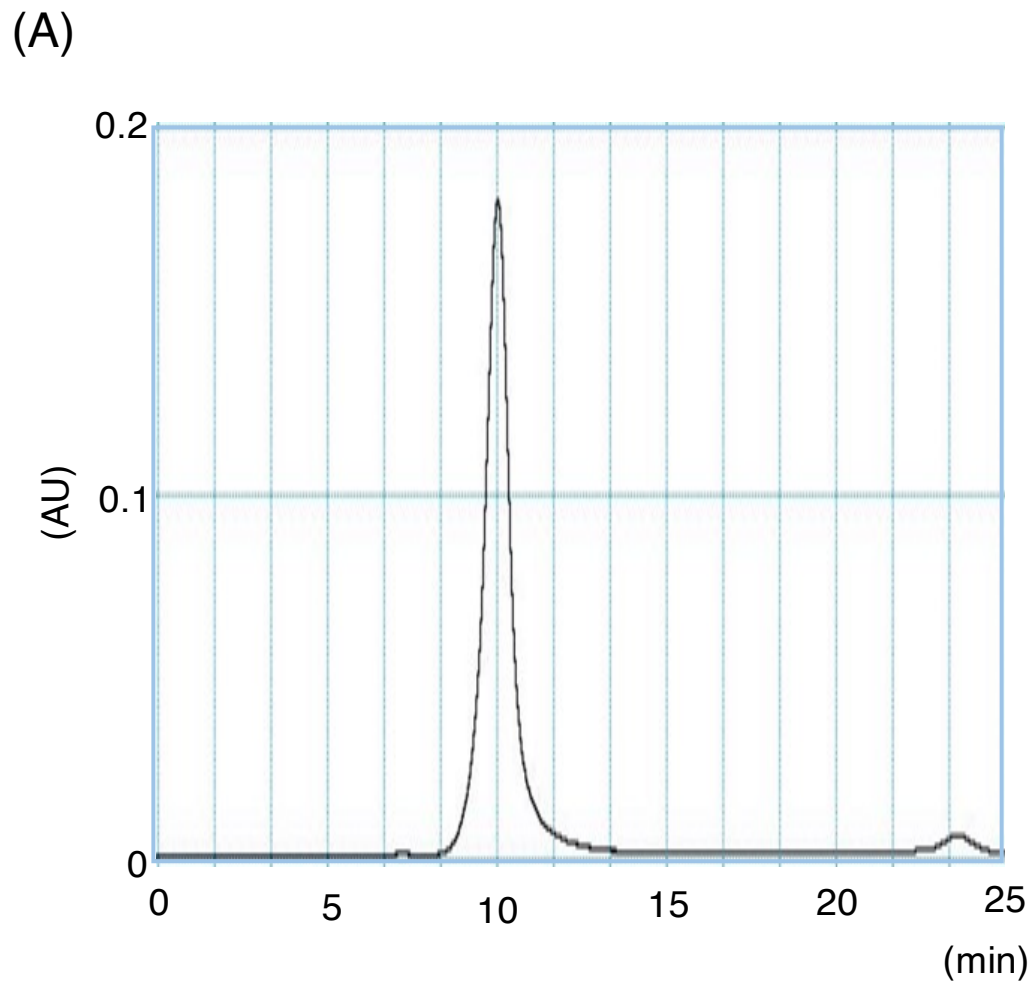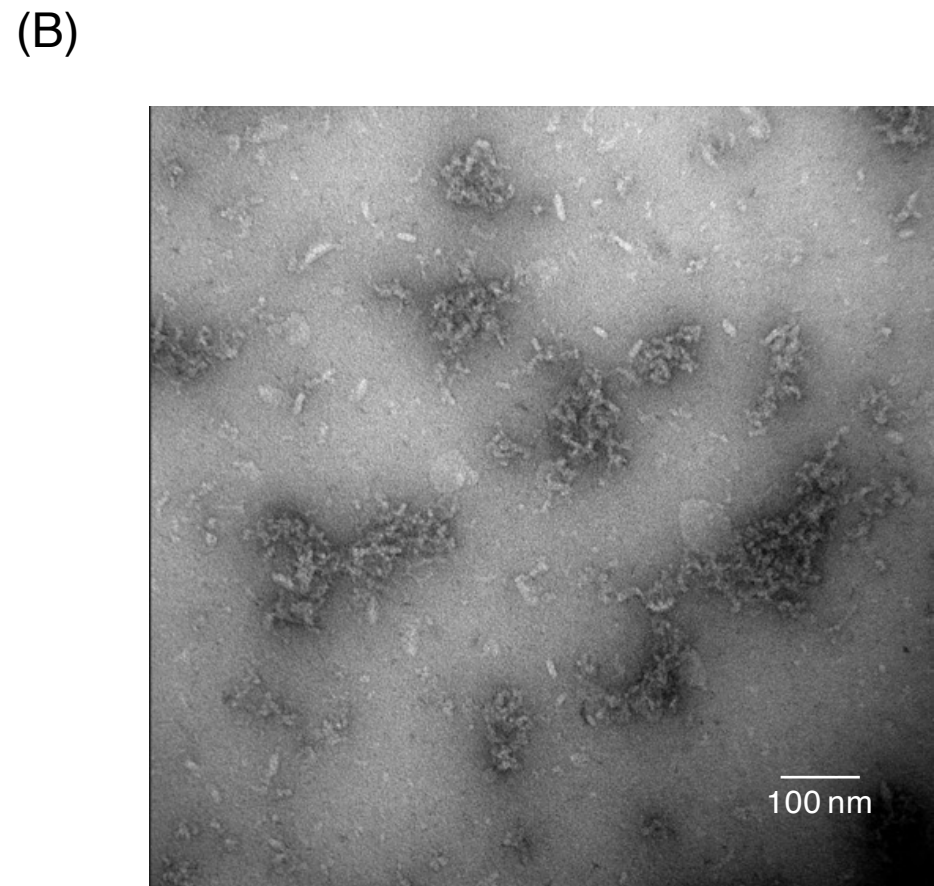

Supplement: Supplementary file 1 [file biomedicines-12-01158-s001.zip › biomedicines-2956198-supplementary.pdf]
